# Habitat potential modelling and the effect of climate change on the current and future distribution of three *Thymus* species in Iran using MaxEnt

**DOI:** 10.1038/s41598-024-53405-5

**Published:** 2024-02-13

**Authors:** Naser Hosseini, Mansour Ghorbanpour, Hossein Mostafavi

**Affiliations:** 1 Department of Medicinal Plants, Faculty of Agriculture and Natural Resources, Arak University, Arak, 38156-8-8349 Iran; 2 Department of Biodiversity and Ecosystem Management, Environmental Sciences Research Institute, Shahid Beheshti University, Tehran, Iran

**Keywords:** Ecology, Evolution, Plant sciences

## Abstract

Over the course of a few decades, climate change has caused a rapid and alarming reshaping of species habitats, resulting in mass extinction, particularly among sensitive species. In order to investigate the effects of climate change on species distribution and assess habitat suitability, researchers have developed species distribution models (SDMs) that estimate present and future species distribution. In West Asia, thyme species such as *T. fedtschenkoi*, *T. pubescens*, and *T. transcaucasicus* are rich in thymol and carvacrol, and are commonly used as herbal tea, spice, flavoring agents, and medicinal plants. This study aims to model the distribution of these Thymus species in Iran using the MaxEnt model under two representative concentration pathways (RCP 4.5 and RCP 8.5) for the years 2050 and 2070. The objective is to identify the crucial bioclimatic (n = 5), edaphic (n = 1), and topographic (n = 3) variables that influence their distribution and predict how their distribution might change under various climate scenarios. The findings reveal that the most significant variable affecting T*. fedtschenkoi* and *T. pubescens* is altitude, while soil organic carbon content is the primary factor influencing the distribution of *T. transcaucasicus*. The MaxEnt modeling demonstrates excellent performance, as indicated by all the area under the curve (AUC) values exceeding 0.9. Based on the projections, it is expected that these three thyme species will experience negative area changes in the coming years. These results can serve as a valuable tool for developing adaptive management strategies aimed at enhancing protection and sustainable utilization in the context of global climate change. Special attention should be given to conserving *T. fedtschenkoi*, *T. pubescens*, and *T. transcaucasicus* due to their significant habitat loss in the future.

## Introduction

Climate change is one of the most important natural factors affecting biodiversity, agricultural production, and food security on a global scale^1–4^. Climate change is accepted by most scientists as a reality, with impacts to plant and animal species forecasted to be complex and dramatic^5–7^. Over the course of a few decades, climate change has rapidly altered the habitats of various species, leading to mass extinction, particularly among sensitive species. This is backed up by studies conducted by e.g. Chhogyel et al., IPCC, Muluneh, Walther, Ruizhi et al. and Khajoei Nasab et al.^8–13^. The basic mechanism about the impacts is that species actually need a number of conditions under which to grow and reproduce successfully, and these conditions are basically determined by abiotic factors, such as soil and climate^14–16^. As climate conditions shift, so too do species’ potential distributions: those areas of the landscape with conditions suitable for a species to persist. The species will become extinct in areas that are no longer suitable, and the extent to which it can migrate and colonize new areas of its potential distribution will define its real distribution^17,18^. Hence, it is crucial to forecast the impact of climate change on plant species, as it plays a critical role in alerting scientists to make informed decisions in the face of future crises. In other words, understanding how species will respond to climate change, including their distribution under future climate change scenarios, is vital for effective management and conservation of biodiversity, as pointed out by Beridze et al.^19^. Several new methods have been developed to explore species distribution patterns based on global warming scenarios^20^. Among them, “species distribution models” (SDMs), which estimate both present and future species distribution, have been extensively developed to investigate the impacts of climate change on species distribution and assess habitat suitability^21^. The maximum entropy model (MaxEnt), which has demonstrated superior performance compared to other models when dealing with limited sample sizes and presence-only data, as shown by Ahmadi et al., Khan et al., and Momeni Damaneh et al. has been widely used to assess ecological requirements, environmental responses, and habitat suitability of species^22–25^.

**Figure 1:**
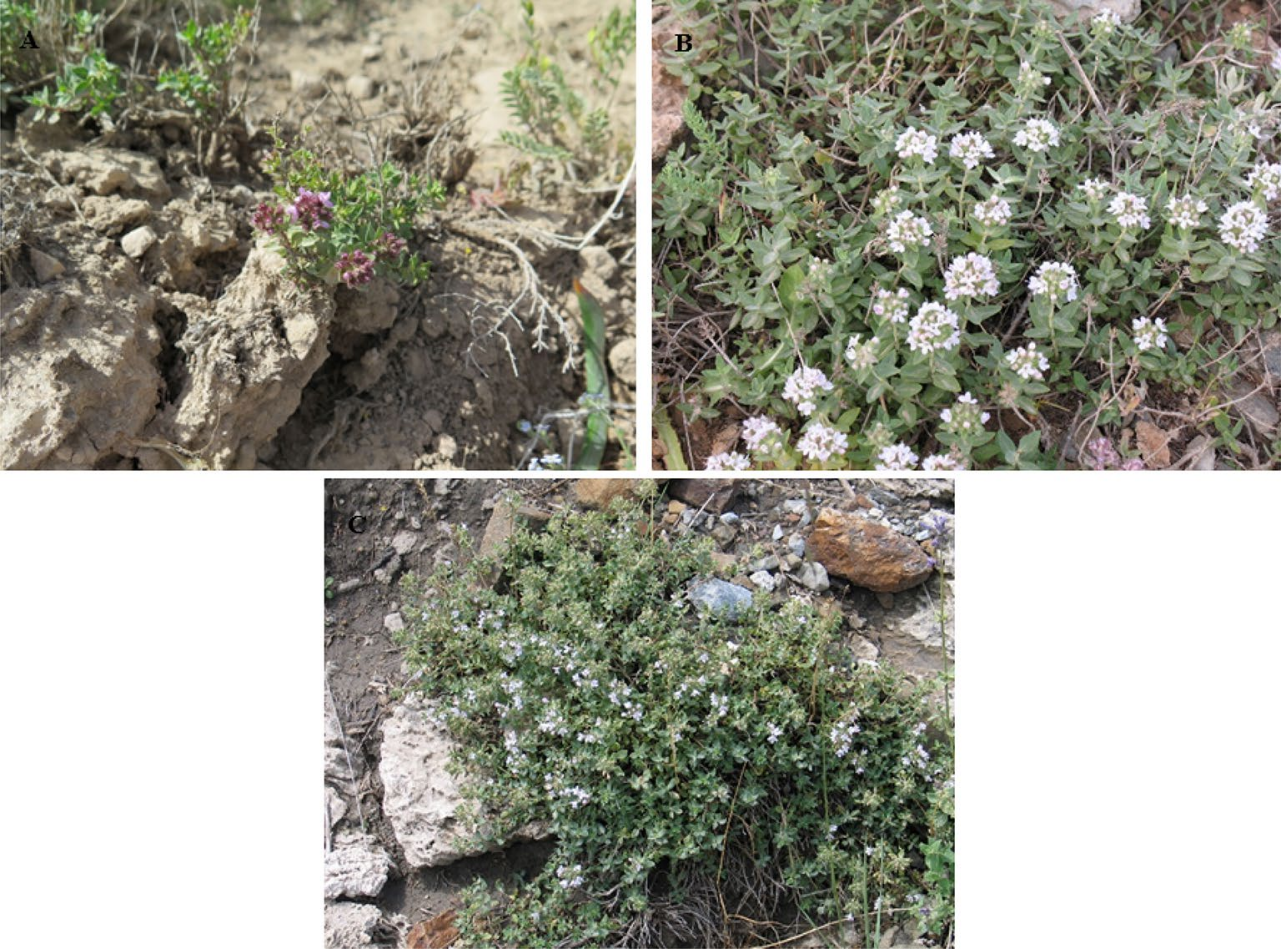
Photographs of *T. fedtschenkoi* (**A**), *T. pubescens* (**B**) and *T. transcaucasicus* (**C**) in Iran.

Iran, with an area of about 1.65 million square kilometers, is a large country located on the Iranian Plateau. It is the second richest country, after Turkey, in terms of plant diversity in the Middle East^26^. Traditional medicine has always been an essential part of Iranian culture and traditions, and many historical books describe Iranian traditional medicine as one of the oldest and richest alternative medicines^27^. Several medicinal plants, such as *Thyme*, are of great significance in traditional medicine and are found within Iran's meadows^28^. The *Thymus* genus, belonging to the Lamiaceae family, includes over 215 species worldwide and is represented in the Iranian flora by 18 species^29–31^. *Thymus* species are mainly concentrated in the western or northern highlands of Iran, such as West Azerbaijan, East Azerbaijan, Ardabil, Zanjan, Kurditan, Mazandaran, Golestan, Tehran, Hamadan, Markazi, and North Khorasan^29,30^. The *Thymus* genus is one of the most popular plants worldwide due to its volatile constituents. Essential oils of *Thymus fedtschenkoi* Ronniger, *Thymus pubescens* Boiss. & Kotschy ex Celak., and *Thymus transcaucasicus* Ronniger (Fig. 1), include high amounts of valuable phenolic compounds such as thymol and carvacrol^32–35^. Understanding how species will adapt to climate change and how they will be distributed in future climate change scenarios is crucial for effective biodiversity management and conservation^36,37^. Although SDMs have been widely used in Iran for various purposes e.g. investigating the future distribution of white mangroves^38^, mapping the habitat suitability of endemic and sub-endemic almond species in Iran^39^, modeling and predicting habitat suitability for *Ferula gummosa*^40^, mapping the current and future distributions of *Onosma* species endemic to Iran^41^, the effect of climate change on the ecological niches of *Bromus tomentellus*^42^, modeling the distribution of some medicinal plant species^43^, and modeling climate change effects on Zagros forests in Iran^44^, no previous study has investigated the impact of climate change on the future distribution of the three valuable *Thymus* species within Iran so far.

The main objectives of the present study are related to the *Thymus* species found in Iran. The study aims to achieve the following goals: (1) Identify the most influential climatic and edaphic variables that affect the distribution of these species. (2) Predict the distribution of *Thymus* spp. under the current climatic and environmental conditions. (3) Estimate the currently suitable distribution areas of the mentioned species within Iran. (4) Forecast the potential distribution variations under proposed future climate change scenarios. (5) Identify species that are vulnerable to the effects of climate change and prioritize their conservation, providing valuable information for decision-makers involved in future conservation planning. By addressing these objectives, the study aims to offer crucial insights and guidance for conservation strategies. This will enable decision-makers to make informed choices concerning the preservation and management of these species in the face of climate change.

## Materials and methods

### Study area

The study area is situated within the geographic boundaries of Iran, covering a total surface area of 1.65 million square kilometers between 44°–64°E latitude and 25°–40°N longitude. Iran, the second-largest country in the Middle East, is located in the arid zone of Asia. It shares its borders with Armenia and the Republic of Azerbaijan to the northwest, Turkmenistan to the northeast, Afghanistan and Pakistan to the east, Turkey and Iraq to the west, and the Persian Gulf and the Gulf of Oman to the south. Iran is an important part of the orogenic belt that encompasses the Asian block, which includes the Zagros, Alborz, and other mountain chains, as well as Arabian-African units. The Zagros, Alborz, Kopet-Dagh, and various interior mountain chains are among the primary geomorphological units in Iran. Damavand peak is Iran’s highest point, towering 5670 m above sea level (m.a.s.l.). The average annual rainfall in Iran is approximately 250 mm, according to Jamshidi and Samani^45^.

### Species occurrence data

**Figure 2:**
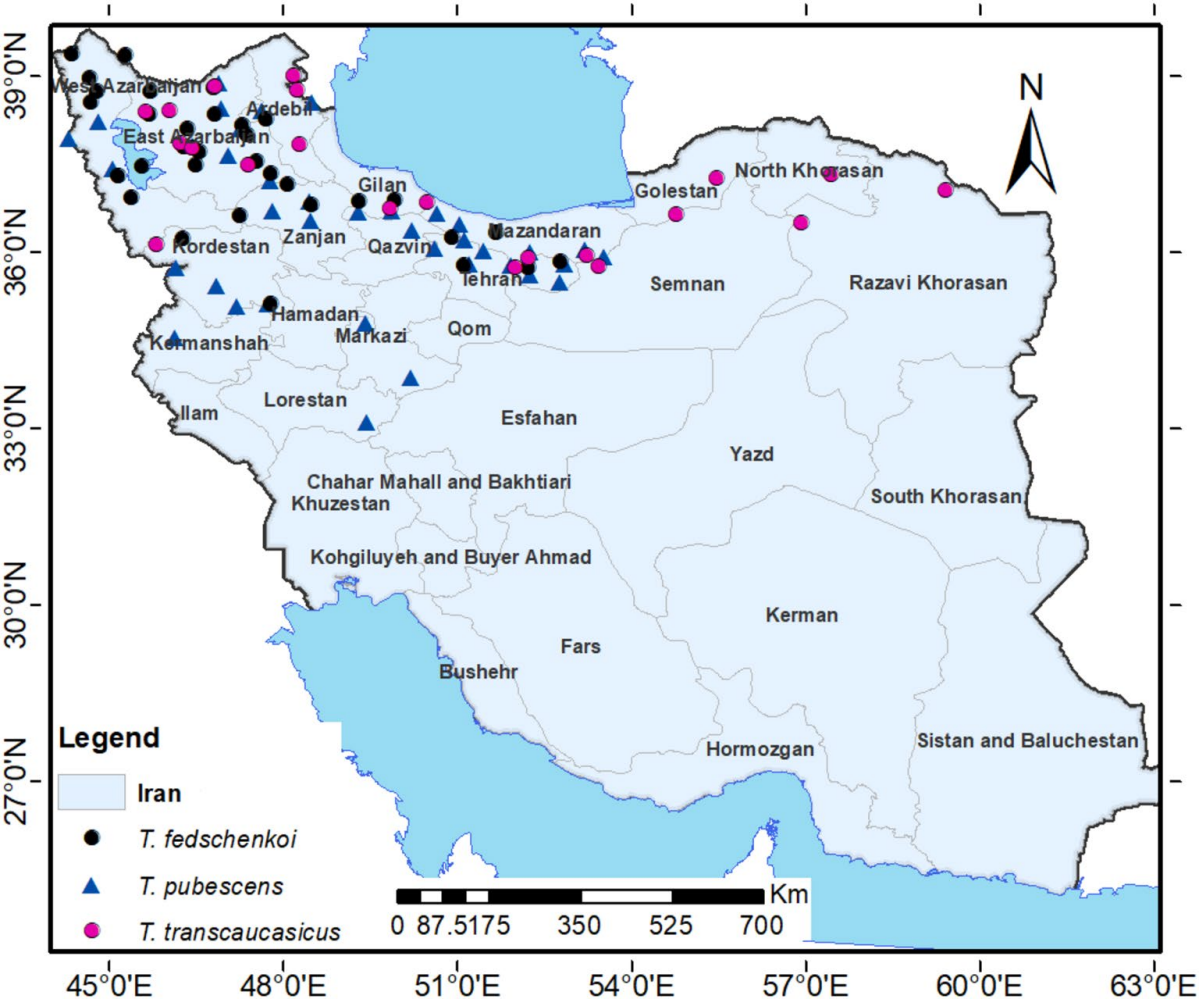
Distribution map (presence data) of the *Thymus* spp. in Iran using Arc-map 10.8.1 software (URL: https://www.arcgis.com/index.html).

We identified the presence points of *Thymus* spp. (Fig. 1) by gathering data from various sources, such as historical records available in the herbaria of HSBU, IRAN and TARI (the acronyms used in the study of Thiers) and literature review^29,30,46^ and www.gbif.org. We then verified all recorded points by conducting extensive fieldwork. As we did not have any reliable absence data for the species distribution, we relied solely on the presence data. Therefore, we generated a comprehensive distribution map that shows the occurrences of the three *Thymus* spp. in Iran, using all available records (Fig. 2).

### Environmental variables

Based on literature reviews and expert opinion^30,47–50^, we have identified three categories of datasets, namely bioclimatic, topographic, and edaphic, which consist of 9 environmental variables that may affect the distribution of *Thymus* spp. (Table 1). We have also assessed the collinearity among these variables using Pearson's correlation coefficient (r), as suggested by previous researchers^24,51–53^. If two variables showed a high correlation (r >|0.70|), we excluded one of them to mitigate collinearity^25^.

**Table 1:** Environmental variables related to the distribution of the *Thymus* spp. in Iran.

Category	Variables	Abbreviation	Unit
Bioclimatic variables (www.worldclim.org)	Mean diurnal range (mean of monthly (max temp—min temp))	Bio2	^o^C
	Mean temperature of driest quarter	bio9	^o^C
	Precipitation of driest month	bio14	mm
	Precipitation seasonality (coefficient of variation)	bio15	mm
	Precipitation of warmest quarter	bio18	mm
Edaphic variables (www.soilgrid.org; www.isric.org)	Soil organic carbon content	Ocr	g kg^–1^
Topographic variables (www.worldgrids.org)	Altitude	Alt	m a.s.l
	Slope	Slope	%
	Solar radiation	Solar	kJ m^–2^ day^–1^

### The distribution modeling procedure

MaxEnt is a Java application that models the present and future habitat suitability of species using species distribution modeling (SDM). It does so with a limited number of records, which are the locations where the species has been found, and environmental variables. The application estimates the distribution or the geographic range of a species by finding the distribution with the maximum entropy, which means it is closest to being geographically uniform, subject to constraints derived from the environmental conditions at the occurrence locations. These constraints are defined in terms of features, which are environmental variables such as temperature, and simple functions of those variables such as quadratic terms. The mean of each feature should match the sample mean. This formulation is equivalent to maximizing the likelihood of a parametric exponential distribution^51^.

MaxEnt version 1.0–3 was used in the R programming environment version 4.3.1 through the "dismo" package version 1.3–9 for modeling purposes (https://rspatial.org/raster/sdm/; Ref.^54^. The evaluation of models was carried out using tenfold cross-validation to estimate errors and ensure consistency. The data were randomly divided into ten parts, out of which nine were used for model fitting while the remaining part was used for evaluation. The trained MaxEnt model's sensitivity and specificity were used to define the proper threshold for predicting the model's output. To predict the future and current suitability, a single MaxEnt model was used with the entire dataset being refitted.

### Model evaluation

In order to evaluate how accurate our model results were, we used a measure called the area under the curve (AUC) of the receiver operating characteristic (ROC) curve. This measure is independent of threshold selection, making it a powerful tool for assessing model performance. The AUC score ranges from 0 to 1.0, with a score of 0.5 indicating random prediction performance and a score of 1.0 indicating perfect performance. Scores below 0.50 indicate that the model is performing worse than random. To determine the primary environmental variables that had an impact on the potential distribution of the studied species under current climate conditions, we used the Jackknife test. We also calculated the percent contribution (PC) of each environmental variable to understand which variables had the biggest impact on potential species distribution. Finally, we used permutation importance (PI) to determine how dependent the model was on each variable.

### Forecasting future distribution

We utilized projected future climate variables for 2050 (averaged for 2041–2060) and 2070 (averaged for 2061–2080) and the average of 16 general circulation models (GCMs) to represent potential distributions of *Thymus* spp. We considered semi-optimistic (RCP4.5) and pessimistic (RCP8.5) greenhouse gas emission scenarios. RCP4.5 is a moderate greenhouse gas emission scenario, while RCP8.5 is the maximum greenhouse gas emission scenario. The higher the greenhouse gas emissions, the higher the global temperatures and the more pronounced the effects of climate change^55^. We standardized the environmental parameters to a common spatial resolution of 30 s of latitude/longitude (approximately 1 km^2^ at ground level)^56^. We used ArcGIS software (Ver 10.2) to create suitability maps for present and future climate scenarios for each species.

## Results

### Evaluations of the model and its importance to variables under current climatic condition

**Figure 3:**
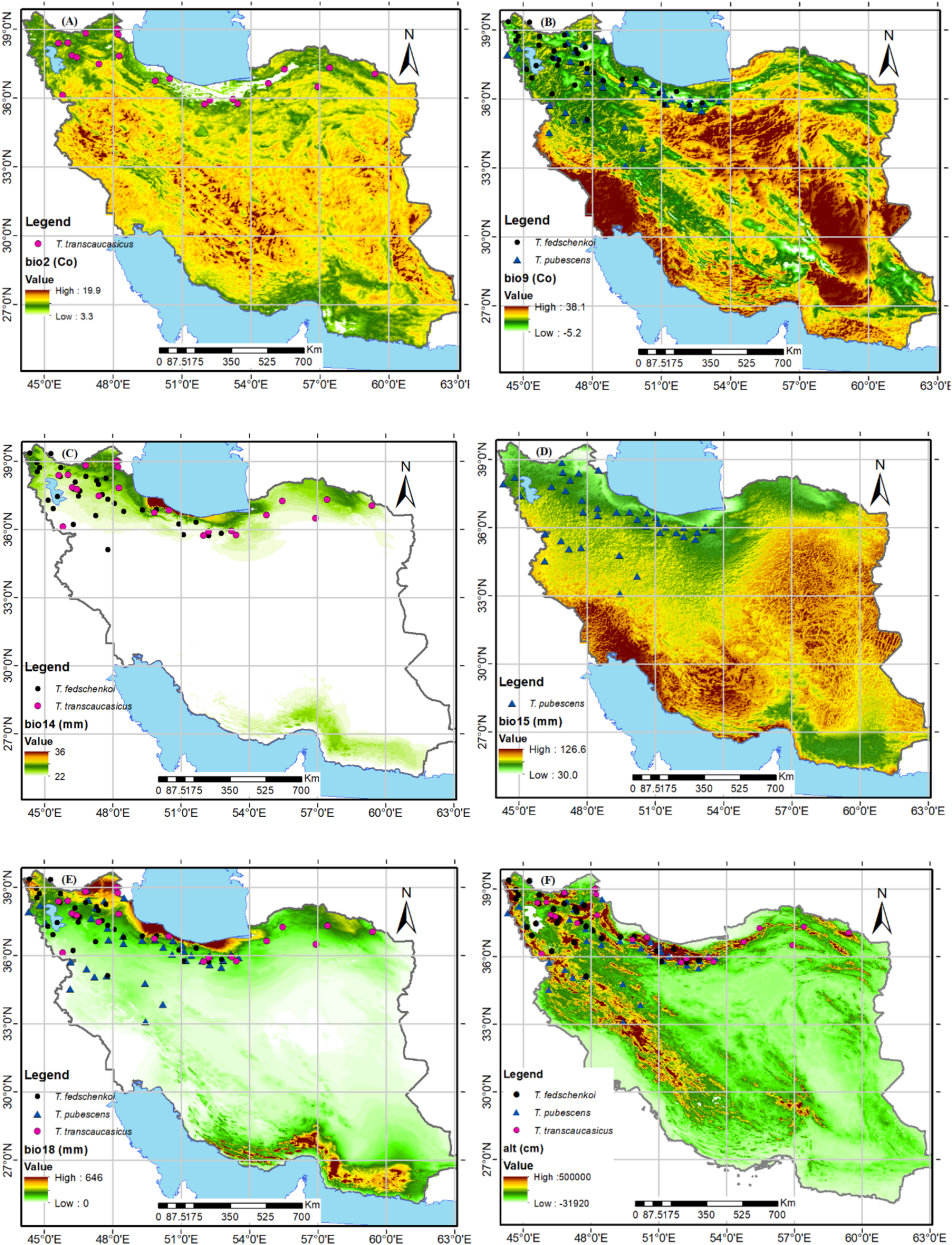

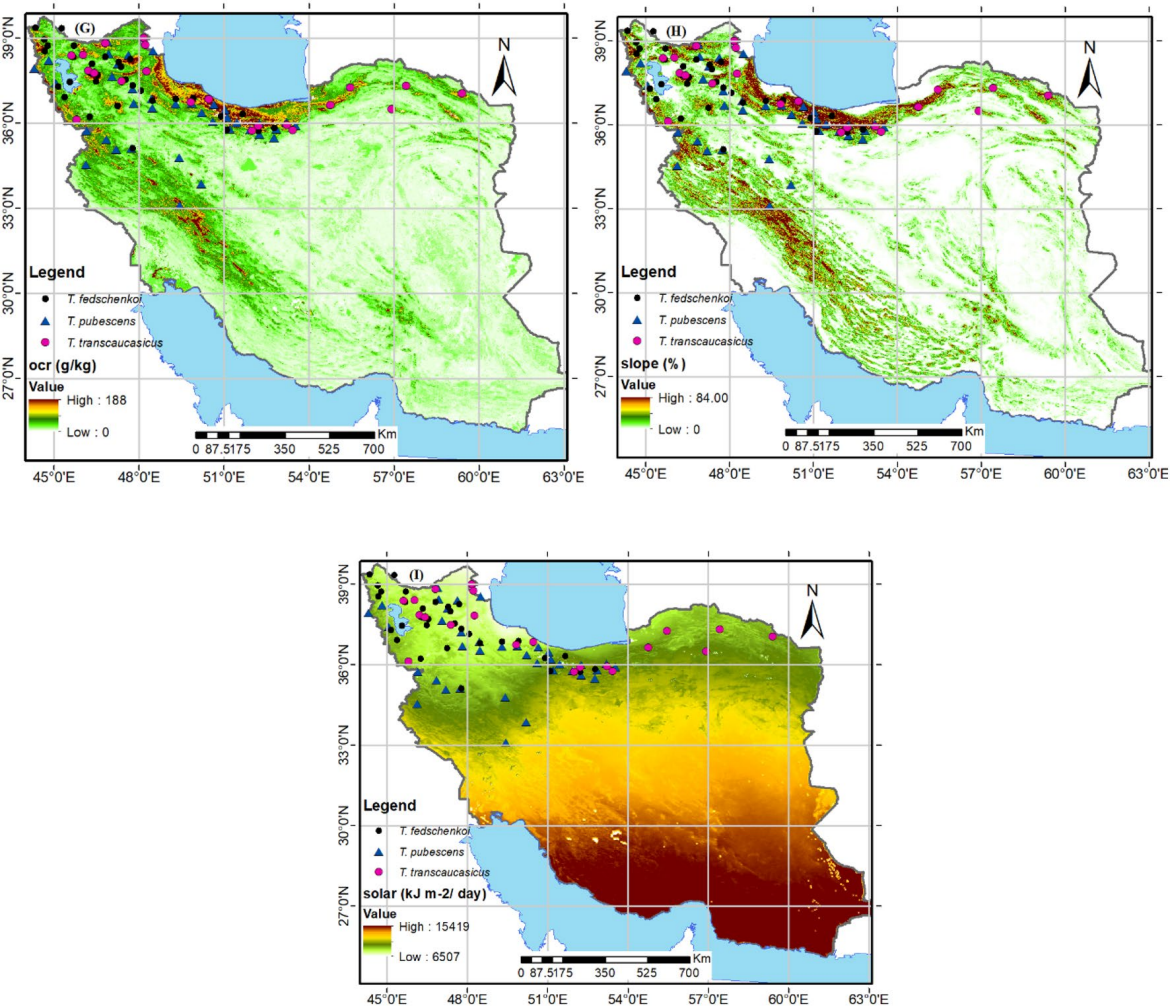
Selected environmental variables for modeling of the *Thymus* spp. using Arc-map 10.8.1 software (URL: https://www.arcgis.com/index.html) Abbreviations are described in Table 1.

The study analyzed the modeling performance for three different species: *T. fedtschenkoi*, *T. pubescens*, and *T. transcaucasicus*. The AUC results revealed that *T. transcaucasicus* had the highest modeling performance (0.975), followed by *T. fedtschenkoi* (0.960) and *T. pubescens* (0.940) (figures not shown). Based on the Pearson’s correlation test, all three species were retained for modeling, and 9 variables were selected for each (Table 2). The importance of these variables varied significantly among the species. For *T. fedtschenkoi*, the most important variables were elevation (33.8%) and precipitation of the driest month (26.2%) (Fig. 3 panels F and C; Table 2). For *T. pubescens*, elevation (47.9%), solar (18.1%), and precipitation of the warmest quarter (10.8%) were the most restrictive environmental variables (Fig. 3 panels F and I; Table 2). Soil organic carbon content (34.6%), precipitation of the driest month (29.7%), and elevation (18.1%) were the most significant factors for *T. transcaucasicus*. Furthermore, some variables were unique to each species, while others were shared. Precipitation seasonality (bio15) was unique to *T. pubescens*, while mean diurnal range (bio2) was unique to *T. transcaucasicus* (Fig. 3 panels D and 2A; Table 2). Mean temperature of the driest quarter (bio9), precipitation of the warmest quarter (bio18), soil organic carbon content, elevation, slope percentage, and solar variables were shared by all three species. It is important to note that some variables were excluded from certain species to avoid collinearity and because their percent contribution varied among the species (Fig. 3). These findings can have significant implications for predicting the distribution and habitat suitability of these species in the future.

**Table 2:** Percent contribution (PC) and permutation importance (PI) of the environmental variables of the *Thymus* spp.

Environmental variable	Description	Species					
		*T. fedschenkoi*		*T. pubescens*		*T. transcaucasicus*	
		PC	PI	PC	PI	PC	PI
Bio2	Mean diurnal range (mean of monthly (max temp—min temp))	–	–	–	–	6.1	48.7
bio9	Mean temperature of driest quarter	2.4	0.4	1.9	5.2	–	–
bio14	Precipitation of driest month	26.2	1.9	–	–	29.7	2.4
bio15	Precipitation seasonality (coefficient of variation)	–	–	6.2	37.4	–	–
bio18	Precipitation of warmest quarter	3	8.9	10.8	0.8	1.2	17.1
Ocr	Soil organic carbon content	5.4	0.8	6.4	0.5	34.6	5
Alt	Altitude	33.8	14.7	47.9	34.2	18.1	20.5
Slope	Slope percentage	14.4	7.6	8.8	11.9	9.9	5.7
Solar	Solar radiation	14.7	65.6	18.1	10.1	0.6	0.5

*Dash (–) denote that some variables do not include for all species.

### Predicted current potential distribution

**Figure 4:**
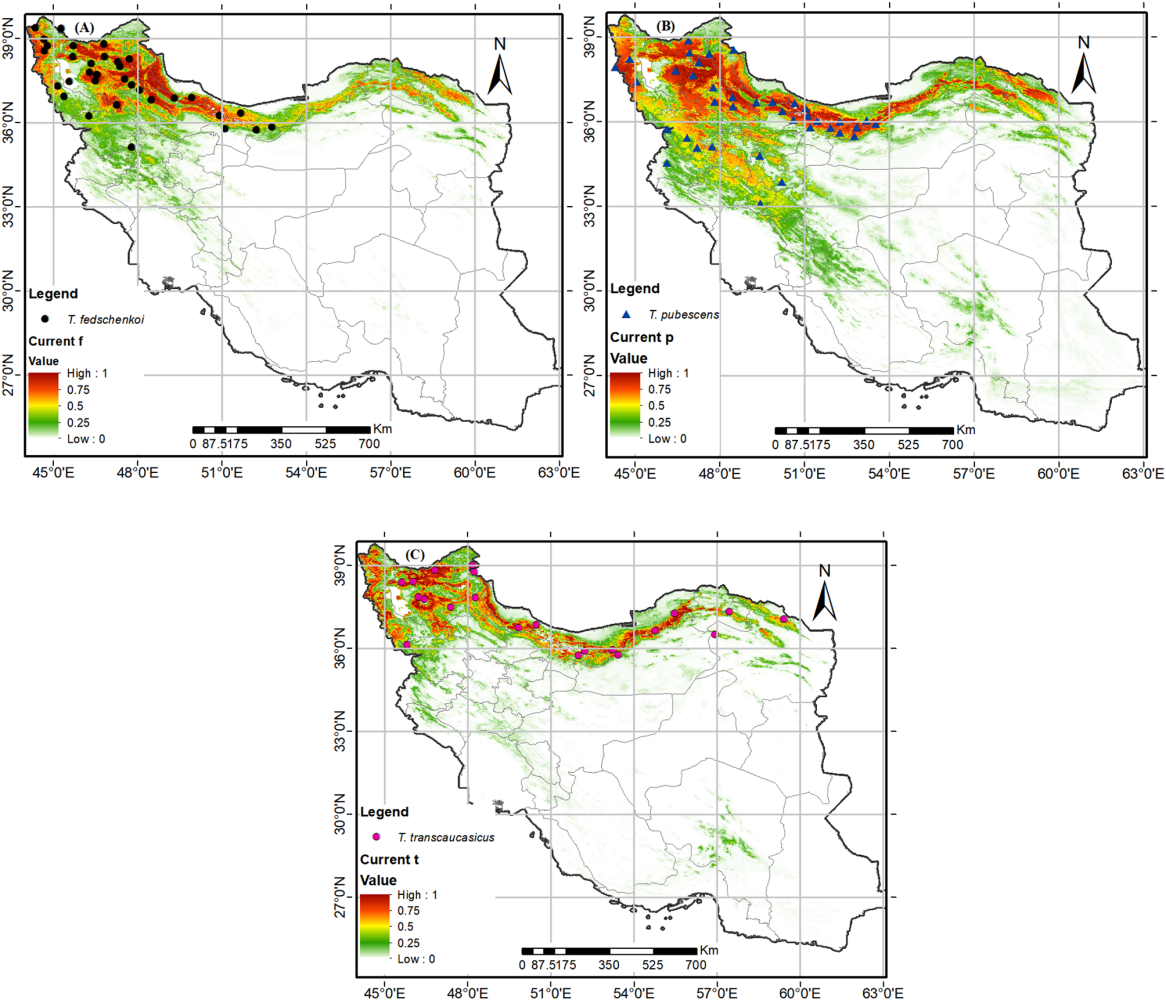
Map for potential current habitat suitability of the *Thymus* spp according to occurrence records in Iran. using R programming environment version 4.3.1 (URL: https://cran.r-project.org/bin/windows/base/).

The MaxEnt projections and current distribution of all species were found to be well-matched in Fig. 3. According to the MaxEnt modeling, the mountains of northwest and north Iran, including West Azarbaijan, Eest Azarbaijan, Ardabil, Qazvin, Zanjan, Tehran, Alborz, Gilan, Mazandaran Golestan and North Khorasan Provinces, are the current most suitable habitats for *T. fedtschenkoi* (Fig. 4A). For *T. pubescens* (Fig. 4B), the mountains of west and northwest and north of Iran, including West Azarbaijan, Eest Azarbaijan, Ardabil, Qazvin, Zanjan, Tehran, Gilan, Mazandran, Golestan, Kordestan, Kermanshah, Lorestan, Esfahan, Hamadan and Markazi Provinces, are the most suitable habitats. Similarly, the most suitable habitats for *T. transcaucasicus* are mountains in the northwest and north of the country, including West Azarbaijan, Eest Azarbaijan, Ardabil, Zanjan, Tehran, Gilan, Mazandaran, Golestan and North Khorasan Provinces. It is worth noting that the current potential distribution of the three species is significantly larger than their actual occurrence, as shown in Fig. 4.

### Predicted future potential distribution

**Figure 5:**
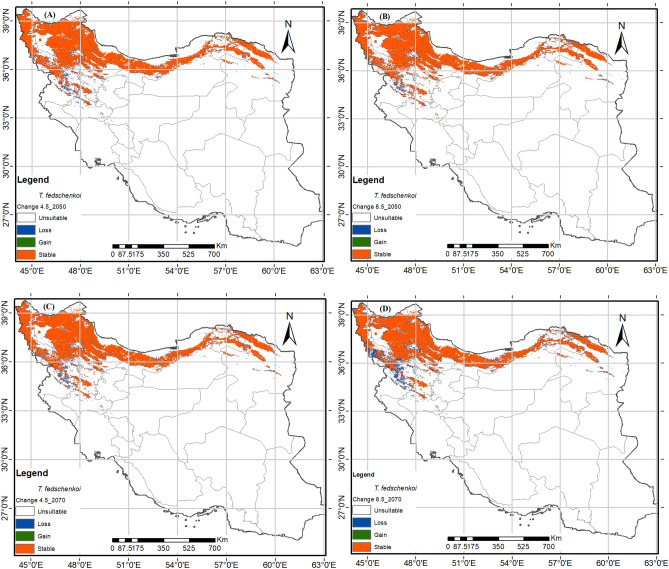
The future potential distribution map of *Thymus fedschenkoi* in 2050 (average for 2041–2060) and 2070 (average for 2061–2080) under semi-optimistic (RCP4.5) and pessimistic (RCP8.5) scenarios. using R programming environment version 4.3.1 (URL: https://cran.r-project.org/bin/windows/base/).

**Figure 6:**
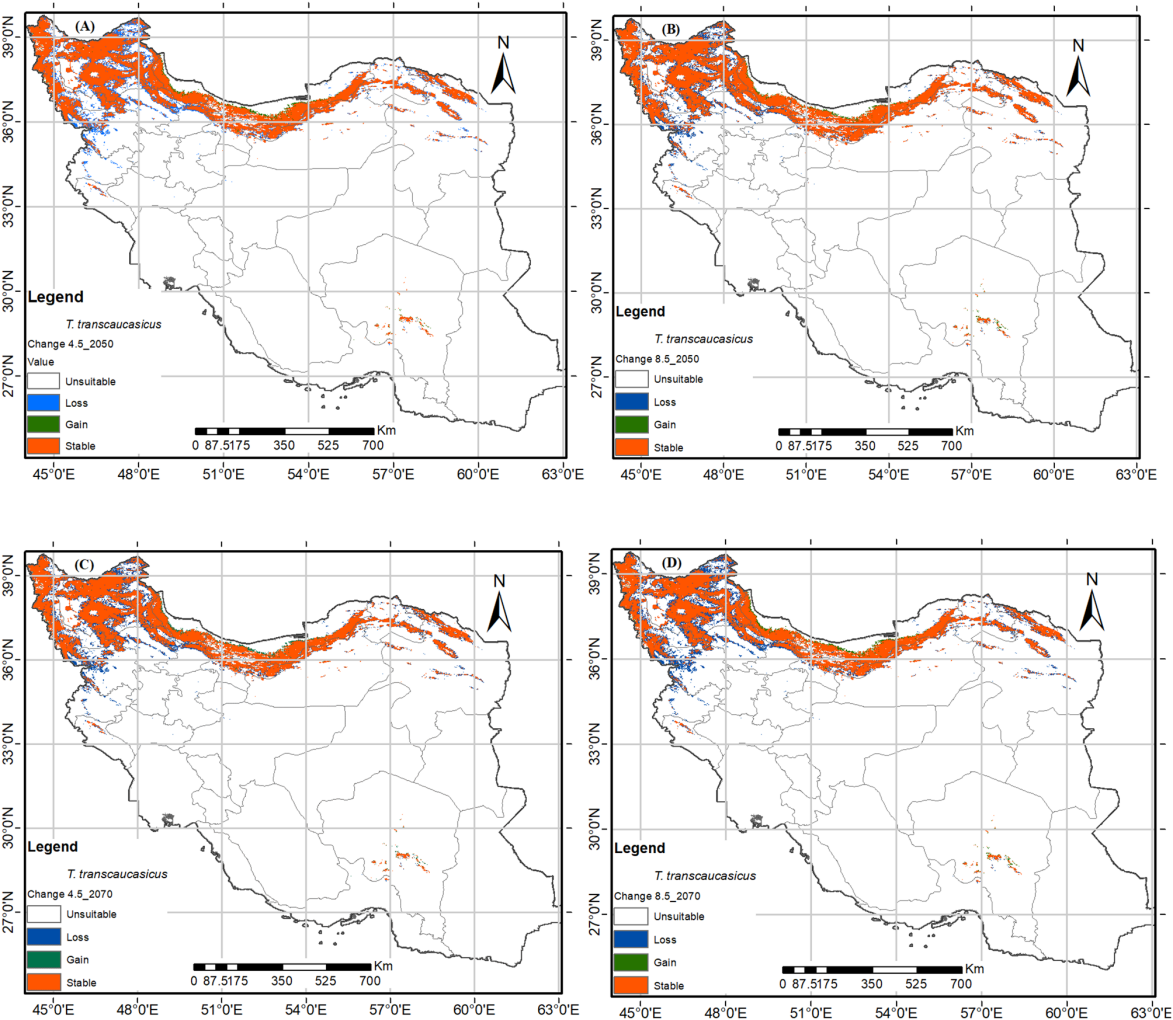
The future potential distribution map of *Thymus transcaucasicus* in 2050 (average for 2041–2060) and 2070 (average for 2061–2080) under semi-optimistic (RCP4.5) and pessimistic (RCP8.5) scenarios. using R programming environment version 4.3.1 (URL: https://cran.r-project.org/bin/windows/base/).

It is evident that each species goes through a gradual transition in time zones and habitats, as shown in Figs. 5, 6. Based on different scenarios, three *Thymus* species are projected to experience negative changes in their range by 2050 and 2070. Figure 5 displays the future potential distribution map of *T. fedschenkoi* under semi-optimistic (RCP4.5) and pessimistic (RCP8.5) scenarios for the years 2050 and 2070. Although the percentage of loss varies (Table 3), T. pubescens, *T. transcaucasicus*, and *T. fedtschenkoi* will encounter the most significant losses under the RCP8.5 scenarios, with reductions exceeding − 29%, − 21%, and − 14%, respectively.

**Table 3:** Percentage of the gain, loss, and range change of the *Thymus* spp. under semi-optimistic (RCP4.5) and pessimistic (RCP8.5) climate change scenarios of 2050 (average for 2041–2060) and 2070 (average for 2061–2080).

Scenario	Time						
	Species	2050			2070		
		Gain (%)	Loss (%)	Change (%)	Gain (%)	Loss (%)	Change (%)
RCP4.5	*T. fedtschenkoi*	0.11	8.82	− 8.71	0.4	8.75	− 8.71
	*T. pubescens*	0.04	22.55	− 22.51	0.08	23.65	− 23.57
	*T. transcaucasicus*	1.78	17.63	− 15.85	1.64	16.37	− 14.72
RCP8.5	*T. fedtschenkoi*	0.02	9.81	− 9.78	0.06	14.66	− 14.60
	*T. pubescens*	0.03	25.57	− 25.54	0.02	29.91	− 29.89
	*T. transcaucasicus*	1.89	19.15	− 17.26	2.5	23.14	− 21.09

**Figure 7:**
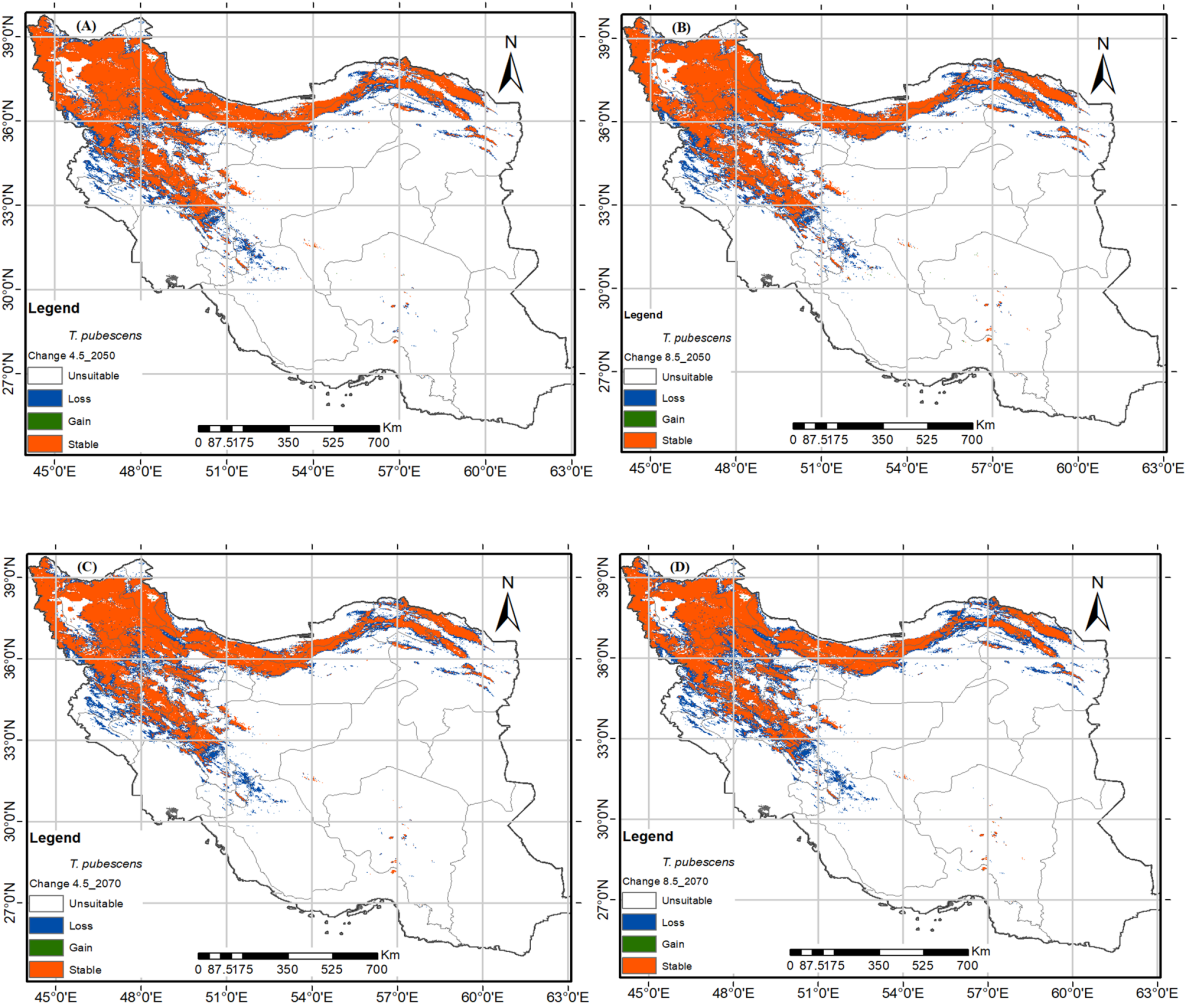
The future potential distribution map of *Thymus pubescense* in 2050 (average for 2041–2060) and 2070 (average for 2061–2080) under semi-optimistic (RCP4.5) and pessimistic (RCP8.5) scenarios. using R programming environment version 4.3.1 (URL: https://cran.r-project.org/bin/windows/base/).

These species will be most affected in the regions of Kordestan and Kermanshah provinces, as shown in Figs. 5, 6. For *T. pubescens*, the distribution range will also decline in lower altitude parts of Isfahan, Lorestan, Golestan, and North Khorasan provinces. Similarly, for *T. transcaucasicus*, the range will decline in Kordestan East Azerbaijan, Ardabil, Zanjan, and North Khorasan Provinces (Fig. 7).

## Discussion

### Environmental variables affecting the distribution of Thymus spp

It is important to understand the environmental factors that influence the distribution of a species from an ecological perspective^57,58^. By conducting a jackknife test and analyzing the variables used in the model, we were able to identify the main environmental factors affecting the distribution of three species of *Thymus* plants. Our predictions showed that the variables that played the biggest role in determining the suitable habitat for each species were as follows: For *T. fedschenkoi*, altitude, precipitation of the driest month (bio14), solar radiation, and slope percentage were the most influential variables. For *T. pubescens*, altitude, solar radiation, and precipitation of the warmest quarter (bio18) were the most important variables. For *T. transcaucasicus*, soil organic carbon content (Ocr), precipitation of the driest month (bio14), and altitude were the most significant factors affecting its distribution. These findings can be further explored in Table 3 and Fig. 3.

According to McCutchan and Fox and Joly et al. an increase in elevation results in a linear decrease in temperature, which has a significant impact on the distribution of living organisms^59,60^. Our study found that three species are distributed in the slopes of the mountainous regions in the Caspian Sea, and Iraniano-Turanian regions, particularly in well-drained soils^31^. Slope percentage plays a vital role in regulating the spatial arrangement of soil nutrients, soil stability^61,62^, water availability, heat, and sunlight^63^. It creates diverse microclimates with unique soil properties, especially in mountainous regions like Iran^24,64,65^. Plant distribution patterns in mountainous areas are influenced by slope percentage, which is recognized as a key topographic factor^66^. In a study conducted in the west and northwest of Iran, it was found that the first species grows in loamy clay-loam soils and requires more precipitation than the second species that grows in sandy-loam soils^48^. Altitude and slope percentage are important ecological variables in the distribution of *Thymus* species including the ones mentioned in this study^49,67,68^.

Corticchiato et al. stated that various factors influence the distribution of *Thymus* species in eastern regions of Spain, including climate, altitude, soil type, soil texture, organic matter (Ocr), and calcium content of soil^69^. Boira and Blanquer also noted that elevation, soil texture, and climate affect the development of *T. piperella* in Spain^70^. Esfanjani et al. found that pH, elevation, precipitation, and temperature variation are the most important environmental factors that affect the distribution of *T. kotschyanus*^71^. The study (Fig. 3; Table 2) suggests that high elevation and low to moderate seasonal precipitation (bio15) are the major factors influencing the distribution range of *T. pubescens*. On the other hand, *T. fedschenkoi* requires summer precipitation (bio14) as well as elevation and slope percentage (as topographic factors). For *T. transcaucasicus*, the amount of soil organic carbon (Ocr) has a greater impact on its distribution than altitude. Precipitation (bio14) is also a significant factor for this species, while elevation has less influence.

### Predict current distribution potential of the *Thymus* spp.

The MaxEnt model is a widely used tool in various fields, including ecology, conservation biology, evolutionary biology, and invasive species management. A study conducted in Iran was the first to investigate the impact of global climate change on the geographical range and suitable habitat of three popular *Thymus* plants, using MaxEnt modeling. This is a crucial step towards devising an effective conservation strategy for these plants. *Thymus* spp. is commonly used for medicinal purposes, including carminative, digestive, antispasmodic, anti-inflammatory, and expectorant effects. The accuracy of the MaxEnt model used in this study was found to be very high, according to the AUC > 0.9 for all species. This indicates that the model's performance was excellent. The MaxEnt model has also been used in other national-scale studies, such as those conducted in Iran, Tunisia, China, and East Asia, to predict potential distributions in various plant species.

The MaxEnt model results have identified the current highly suitable habitats for *T. fedschenkoi* mostly located in the northwest and north of the country, as shown in Fig. 3, panel A. These regions are distinguished by an elevation of 1400–3500 m, precipitation of driest month more than 10 mm, and high slope percentage greater than 20%. Autecology studies of *T. fedschenkoi*, conducted by Ghelichnia at an altitude of Mazandaran province in Iran, have revealed that the suitable distribution altitude range of the mentioned species was from sea level to 1400 m.a.s.l. Our findings are in line with the mentioned studies. The mentioned species is distributed in areas with moderate to high slopes, and the precipitation of the driest month (bio14) plays a crucial role in the dispersion of this species^32^.

Based on the suitable habitat modeling, *T. pubescens* is found in the west, north, and northeast regions of the country (as shown in Fig. 3B). Despite this, the actual range of the species does not include the northeast region. These areas are characterized by low solar radiation (< 6000 kJ m^–2^ day^–1^), high slope percentage (> 30%), and an altitude of more than 1500 m.a.s.l. The research on this species revealed that it grows well in rocky slopes of the Irano-Turanian and Hyrcanian mountains, where it can reach elevations of up to 3000 m. These mountains have calcareous sandy loam soils and a cold, dry climate. According to Fig. 3, regions with high summer rainfall are not suitable for this species. Therefore, areas like the Caspian Sea coast, which have low elevation and high summer precipitation, are not suitable for this species' dispersal. On the other hand, the MaxEnt model results show that the majority of the country's current suitable habitats for *T. transcaucasicus* are located in the northwest, north, and northeast regions (as displayed in Fig. 3C). These regions are characterized by low soil organic carbon content, high rainfall in the driest month, and an altitude of less than 2000 m.a.s.l. This species is more moisture friendly and can grow at lower elevations than *T. pubescens*^72^. Therefore, it is expected to find this species in highland areas near the Caspian Sea and its absence in lower latitudes with lower precipitation.

### Predicted potential distribution of the *Thymus* spp on the future

There is mounting evidence that the average global temperature is increasing and precipitation is decreasing, partly due to the rise in greenhouse gas emissions^73^. The research and assessments conducted on climate change predict that if the concentration of CO2 doubles by 2100, the average temperature in Iran will rise by 1.5–4.5 °C. The adverse effects of climate change include changes in precipitation and temperature patterns, water resources, sea levels and coastal zones, agriculture and food production, forestry, drought frequency and intensity, and human health^74^. Climate change has also affected the distribution of various species, although the effects vary depending on the species^41,62^. Mountainous plant communities around the world have been found to be diverse^75,76^. As a result, species are shifting their spatial distribution to higher elevations to take advantage of higher precipitation and cooler temperatures, resulting in better-adapted plant growth. Previous research, particularly in mountain ecosystems in Iran and around the world, has also shown an upward shift in plant community distribution under climate change scenarios^43,76,77^. The MaxEnt model indicates that the suitable habitats for the three *Thymus* species will decrease in 2050 and 2070 under semi-optimistic (RCP4.5) and pessimistic (RCP8.5) scenarios (Table 3). *T. pubescens* and *T. fedtschenkoi* experienced the highest (29.89%) and lowest (14.60%) decreases in distribution under RCP8.5–2070, respectively.

*Thymus* species have a limited ecological niche and do not prefer marginal habitats^49^. The studied species lose their habitats in the foothills, particularly in lower latitudes, but the highland habitats remain suitable. According to the modeling results, among the mentioned species, only *T. transcaucasicus* acquires narrow new habitats on the northern slopes in the south of the Caspian Sea in the future. This species requires moderate to high precipitation of the driest month (bio14) (Fig. 3C). Based on studies by Sharifi Ashoorabadi et al. the highest germination percentage of thyme seeds occurs at temperatures ranging from − 4 to 4 degrees. However, at temperatures ranging from − 8 to more than 4, the germination ratio decreases significantly^78^. Furthermore, studies have shown that high humidity reduces the percentage of seed germination, especially when combined with high temperatures. *Thymus* species are most common in cool to moderate annual temperatures in elevated areas^79^. So, climate changes have a negative effect on the distribution range of thyme by having a negative impact on seed germination. The results of this study showed that the adaptation level of real and prediction maps differs regarding the habitat of plant species. The methods based on the presence of plant species have good predictive power and a wide range of ecological nests. These methods are influenced by: (1) the nature of the modeling method, (2) the modeling algorithm, and (3) the characteristics of plant species, including the geographical range of distribution and the range of plant species tolerance to environmental conditions.

### Research limitations

It is important to acknowledge that our study has several limitations. Firstly, the set of 9 environmental variables selected may not cover all the factors that affect the geographic distribution of *Thymus* spp. Other factors such as species interactions, overgrazing, land use change and distance from streams can also play a crucial role in determining the distribution of *Thymus* species. Land use changes can also increase the extent of unsuitable habitats. Despite our study indicating that *T. pubescense* has a wide distribution area, much of the suitable area may disappear due to human activities. Therefore, it is essential to conduct further research to verify the impact of human activities on the adaptation of *Thymus* spp. to future climates. It is also important to note that species distribution models rely on observed species occurrence data to estimate the environmental conditions suitable for that species. However, it is crucial to understand that the predicted suitable habitat area from the model may not always perfectly match the actual habitat where the species is found. This discrepancy can occur due to uncertainties and internal assumptions within the species distribution models. Therefore, further research is necessary to improve the accuracy of predictions by taking into account these factors.

## Conclusion

*Thymus* oils and extracts are widely used in the pharmaceutical, perfume, and cosmetic industries, as well as in the food industry to enhance and protect food products. A recent study used the MaxEnt modeling technique to identify suitable areas for three *Thymus* species and to investigate the impact of climate change on these plants in Iran. The study found that both climate change scenarios (RCP4.5 and RCP8.5) indicate a decrease in suitable areas for these species. This highlights the urgent need for conservation efforts to protect these species, particularly *T. pubescense*, which is projected to lose a significant portion of its habitat in the future. To safeguard these valuable species, continuous monitoring efforts are crucial, and the establishment of new protected areas should be considered based on the projected suitable distribution areas for these species in the future. Taking proactive conservation measures now will be essential to preserve plants and their habitats in the face of changing climatic conditions.

## Data Availability

All the data generated/ analyzed during the study are available with the corresponding author on reasonable request.

## References

[1] Lavergne S, Mouquet N, Thuiller W, Ronce O (2010). Biodiversity and climate change: Integrating evolutionary and ecological responses of species and communities. Annu. Rev. Ecol. Evol. Syst..

[2] Vermeulen SJ, Campbell BM, Ingram JSI (2012). Climate change and food systems. Annu. Rev. Environ. Resour..

[3] Wiebe K, Robinson S, Cattaneo A (2019). Climate change, agriculture and food security: Impacts and the potential for adaptation and mitigation. Sustain. Food Agric..

[4] Fan Z (2022). Impacts of climate change on species distribution patterns of Polyspora sweet in China. Ecol. Evol..

[5] Renton M, Shackelford N, Standish RJ (2012). Habitat restoration will help some functional plant types persist under climate change in fragmented landscapes. Glob. Chang. Biol..

[6] Tovar C (2022). Understanding climate change impacts on biome and plant distributions in the Andes: Challenges and opportunities. J. Biogeogr..

[7] Makki T (2023). Impacts of climate change on the distribution of riverine endemic fish species in Iran, a biodiversity hotspot region. Freshw. Biol..

[8] Chhogyel N, Kumar L, Bajgai Y, Jayasinghe LS (2020). Prediction of Bhutan’s ecological distribution of rice (*Oryza*
*sativa* L.) under the impact of climate change through maximum entropy modelling. J. Agric. Sci..

[9] IPCC, C. C. The physical science basis (2013).

[10] Muluneh MG (2021). Impact of climate change on biodiversity and food security: A global perspective—A review article. Agric. Food Secur..

[11] Walther G-R (2003). Plants in a warmer world. Perspect. Plant Ecol. Evol. Syst..

[12] Ruizhi H (2021). Prediction of suitable distribution area of the endangered plant *Acer*
*catalpifolium* under the background of climate change in China. J. Beijing For. Univ..

[13] Khajoei Nasab F, Mehrabian A, Mostafavi H, Neemati A (2022). The influence of climate change on the suitable habitats of Allium species endemic to Iran. Environ. Monit. Assess..

[14] Krebs CJ (2001). Ecology the Experimental Analysis of Distribution and Abundance.

[15] Peterson AT (2002). Future projections for Mexican faunas under global climate change scenarios. Nature.

[16] Legg S (2021). IPCC, 2021: Climate change 2021-the physical science basis. Interaction.

[17] Makki T (2023). Predicting climate heating impacts on riverine fish species diversity in a biodiversity hotspot region. Sci. Rep..

[18] Zuo J (2023). Analysis of niche shift and potential suitable distributions of Dendrobium under the impact of global climate change. Environ. Sci. Pollut. Res..

[19] Beridze B (2023). Biodiversity protection against anthropogenic climate change: Conservation prioritization of *Castanea*
*sativa* in the South Caucasus based on genetic and ecological metrics. Ecol. Evol..

[20] Rodríguez JP, Brotons L, Bustamante J, Seoane J (2007). The application of predictive modelling of species distribution to biodiversity conservation. Divers. Distrib..

[21] Phillips SJ (2009). Sample selection bias and presence-only distribution models: Implications for background and pseudo-absence data. Ecol. Appl..

[22] Ahmadi M, Hemami M, Kaboli M, Shabani F (2023). MaxEnt brings comparable results when the input data are being completed; Model parameterization of four species distribution models. Ecol. Evol..

[23] Khan AM (2022). MaxEnt modelling and impact of climate change on habitat suitability variations of economically important Chilgoza Pine (*Pinus*
*gerardiana* Wall.) in South Asia. Forests.

[24] Momeni Damaneh J (2022). Prediction of wild pistachio ecological niche using machine learning models. Ecol. Inform..

[25] Elith J (2011). A statistical explanation of MaxEnt for ecologists. Divers. Distrib..

[26] Jafari SM, Akhani H (2008). Plants of Jahan Nama protected area, Golestan province. N. Iran. Pak. J. Bot..

[27] Ameri A (2015). Medicinal plants contain mucilage used in traditional Persian medicine (TPM). Pharm. Biol..

[28] Nobakht SZ, Akaberi M, Mohammadpour AH, Moghadam AT, Emami SA (2022). *Hypericum*
*perforatum*: Traditional uses, clinical trials, and drug interactions. Iran. J. Basic Med. Sci..

[29] Jalas, J. Thymus in KH Rechinger (Ed.), Fl. Iran. 150, 532–551 (1982).

[30] Jamzad Z (2012). Flora of Iran (Lamiaceae).

[31] Ghasemi Pirbalouti A, Emami Bistghani Z, Malekpoor F (2015). An overview on genus Thymus. J. Med. Herbs.

[32] Ghelichnia H (2018). Essential oil composition of *Thymus*
*fedtschenkoi* Ronniger at different growing altitudes in Mazandaran, Iran. Cercet. Agron. Mold..

[33] Ghelichnia H (2016). Essential oil composition of three species of thymus growing wild in Mazandaran, Iran. Cercet. Agron. Mold..

[34] Nickavar B, Malekitabar E (2022). Compositional analysis and antioxidant activities of *Thymus*
*pubescens* essential oil from Iran. Comb. Chem. High Throughput Screen..

[35] Tohidi B, Rahimmalek M, Arzani A (2017). Essential oil composition, total phenolic, flavonoid contents, and antioxidant activity of Thymus species collected from different regions of Iran. Food Chem..

[36] Naudiyal N (2021). Potential distribution of Abies, Picea, and Juniperus species in the sub-alpine forest of Minjiang headwater region under current and future climate scenarios and its implications on ecosystem services supply. Ecol. Indic..

[37] Shi X (2023). Habitat distribution pattern of rare and endangered plant *Magnolia*
*wufengensis* in China under climate change. Forests.

[38] Asgarian A, Soffianian A (2023). Past and potential future distribution of white mangroves in an arid estuarine environment: Integration of Maxent and CA-Markov models. Mar. Policy.

[39] Zeraatkar A, Khajoei Nasab F (2023). Mapping the habitat suitability of endemic and sub-endemic almond species in Iran under current and future climate conditions. Environ. Dev. Sustain..

[40] Mohammady M (2021). Modeling and prediction of habitat suitability for *Ferula*
*gummosa* medicinal plant in a mountainous area. Nat. Resour. Res..

[41] Khajoei Nasab F, Mehrabian A, Mostafavi H (2020). Mapping the current and future distributions of Onosma species endemic to Iran. J. Arid Land.

[42] Bazrmanesh A, Tarkesh M, Bashari H, Poormanafi S (2019). Effect of climate change on the ecological niches of the climate of *Bromus*
*tomentellus* Boiss using Maxent in Isfahan province. J. Range Watershed Manag..

[43] Behmanesh B, Tabasi E, Fakhireh A, Khalasi Ahvazi L (2019). Modeling the distribution of medicinal plant species of *Thymus*
*kotschyanus* and *Achillea*
*millefolium* using ENFA and logistic regression. J. Plant Ecosyst. Conserv..

[44] Valavi R (2019). Modelling climate change effects on Zagros forests in Iran using individual and ensemble forecasting approaches. Theor. Appl. Climatol..

[45] Jamshidi, Z. & Samani, N. Mapping the spatiotemporal diversity of precipitation in Iran (2021).

[46] Thiers, B. Index Herbariorum: A global directory of public herbaria and associated staff. New York Garden’s Virtual Herbarium. New York Gard. Virtual Herb. http//sweetgum.nybg.org/ih (2022).

[47] Asaadi AM, Khoshnod Yazdi A (2016). The autecological characteristics of *Thymus*
*transcaspicus* Klokov medicinal plant in North East Rangelands of Iran. J. Med. Plants.

[48] Najafzadeh R, Rashidi Z, Shokri B, Abdi H (2020). Investigation of morphological and ecological and essential oil content variation of some populations of thyme species (*Thymus* spp.) in the northwest and west of Iran. Iran. J. Rangel. For. Plant Breed. Genet. Res..

[49] Zare Chahouki MA, Abasi M (2016). Habitat suitability modeling for *Thymus*
*kotschyanus* Boiss. & Hohen. using ecological-niche factor analysis (case study: Rangeland of middle Taleghan). Iran. J. Med. Aromat. Plants Res..

[50] Nazari S, Jafarian Z, Alavi J, Naghi Poor AA (2021). The impact of climate change on the geographic distribution of *Thymus Kotschyanus* (Boiss and Hohen) using ensemble modelling. Desert Manag..

[51] Elith J (2006). Novel methods improve prediction of species’ distributions from occurrence data. Ecography (Cop.).

[52] Wu Y-M (2021). Impact of past and future climate change on the potential distribution of an endangered montane shrub *Lonicera*
*oblata* and its conservation implications. Forests.

[53] Yang M (2023). Predicting the potential geographical distribution of Rhodiola L. in China under climate change scenarios. Plants.

[54] Hijmans RJ, Phillips S, Leathwick J, Elith J, Hijmans MRJ (2017). Package ‘dismo’. Circles.

[55] Moss, R. H. et al.* Towards new scenarios for analysis of emissions, climate change, impacts, and response strategies* (2008).

[56] Soilhi Z, Sayari N, Benalouache N, Mekki M (2022). Predicting current and future distributions of *Mentha*
*pulegium* L. in Tunisia under climate change conditions, using the MaxEnt model. Ecol. Inform..

[57] Mirinejad S (2018). Investigating the impact of some habitat characteristics on distribution of *Stachys*
*pilifera* benth using the BMLR model in Iran. Polish J. Environ. Stud..

[58] Yan X (2021). Current and future distribution of the deciduous shrub *Hydrangea*
*macrophylla* in China estimated by MaxEnt. Ecol. Evol..

[59] McCutchan MH, Fox DG (1986). Effect of elevation and aspect on wind, temperature and humidity. J. Appl. Meteorol. Climatol..

[60] Joly D, Castel T, Pohl B, Richard Y (2018). Influence of spatial information resolution on the relation between elevation and temperature. Int. J. Climatol..

[61] Abdi E, Saleh HR, Majnonian B, Deljouei A (2019). Soil fixation and erosion control by *Haloxylon*
*persicum* roots in arid lands, Iran. J. Arid Land.

[62] Deljouei A (2023). Implications of hornbeam and beech root systems on slope stability: From field and laboratory measurements to modelling methods. Plant Soil.

[63] Hao J, Chu LM (2022). Responses of terrestrial bryophytes to simulated climate change in a secondary evergreen broad-leaved forest in Southern China. J. For. Res..

[64] Douaihy CB, Restoux G, Machon N, Dagher-Kharrat MB (2013). Ecological characterization of the *Juniperus*
*excelsa* stands in Lebanon. Ecol. Mediterr..

[65] Santos X (2006). Inferring habitat-suitability areas with ecological modelling techniques and GIS: A contribution to assess the conservation status of *Vipera*
*latastei*. Biol. Conserv..

[66] Oke OA, Thompson KA (2015). Distribution models for mountain plant species: the value of elevation. Ecol. Model..

[67] Darvishi L, Zare Chahouki MA, Jafari M, Azarnivand H, Yousefi Valikchali M (2013). Study on the environmental factors contributing to distribution of *Thymus*
*kotschyanus* in Taleghan Basin, Iran. J. Rangel. Sci..

[68] Pirbalouti AG, Karimi A, Yousefi M, Enteshari S, Golparvar AR (2011). Diversity of *Thymus*
*daenensis* Celak in central and west of Iran. J. Med. Plants Res..

[69] Corticchiato M, Tomi F, Bernardini AF, Casanova J (1998). Composition and infraspecific variability of essential oil from *Thymus*
*herba*
*barona* Lois. Biochem. Syst. Ecol..

[70] Boira H, Blanquer A (1998). Environmental factors affecting chemical variability of essential oils in *Thymus*
*piperella* L. Biochem. Syst. Ecol..

[71] Esfanjani J (2021). Application of modeling techniques for the identification the relationship between environmental factors and plant species in rangelands of Iran. Ecol. Inform..

[72] Tabad MA, Jalilian N, Maroofi H (2016). Study of flora, life form and chorology of plant Species in Zarivar Region of Marivan, Kurdistan. Taxon. Biosyst..

[73] Muñoz AR, Márquez AL, Real R (2013). Updating known distribution models for forecasting climate change impact on endangered species. PLoS One.

[74] Amiri MJ, Eslamian SS (2010). Investigation of climate change in Iran. J. Environ. Sci. Technol..

[75] Hamid M (2019). Impact of climate change on the distribution range and niche dynamics of Himalayan birch, a typical treeline species in Himalayas. Biodivers. Conserv..

[76] Khwarahm NR (2020). Mapping current and potential future distributions of the oak tree (*Quercus*
*aegilops*) in the Kurdistan Region, Iraq. Ecol. Process..

[77] Naghipour Borj AA, Ostovar Z, Asadi E (2019). The influence of climate change on distribution of an endangered medicinal plant (*Fritillaria*
*Imperialis* L.) in central Zagros. J. Rangel. Sci..

[78] Sharifi Ashoorabadi E, Mackizadeh Tafti M, Hasani J, Lebaschy MH (2021). Effect of temperature and humidity on seed germination of six different Thymus species. Iran. J. Seed Sci. Technol..

[79] Yousefzadeh S, Abedi R, Mokhtassi-Bidgoli A (2021). Which environmental factors are more important for geographic distributions of Thymus species and their physio-morphological and phytochemical variations?. Arab. J. Geosci..

